# Monolithic Composite “Pressure + Acceleration + Temperature + Infrared” Sensor Using a Versatile Single-Sided “SiN/Poly-Si/Al” Process-Module

**DOI:** 10.3390/s130101085

**Published:** 2013-01-16

**Authors:** Zao Ni, Chen Yang, Dehui Xu, Hong Zhou, Wei Zhou, Tie Li, Bin Xiong, Xinxin Li

**Affiliations:** State Key Laboratory of Transducer Technology, Shanghai Institute of Microsystem and Information Technology, Chinese Academy of Sciences, 865 Changning Road, Shanghai 200050, China; E-Mails: nizao@mail.sim.ac.cn (Z.N.); dehuixu@mail.sim.ac.cn (D.X.); zhouhong@mail.sim.ac.cn (H.Z.); zhouwei@mail.sim.ac.cn (W.Z.); tli@mail.sim.ac.cn (T.L.); bxiong@mail.sim.ac.cn (B.X.)

**Keywords:** composite sensor, sensing networks, MEMS, single-sided, process

## Abstract

We report a newly developed design/fabrication module with low-cost single-sided “low-stress-silicon-nitride (LS-SiN)/polysilicon (poly-Si)/Al” process for monolithic integration of composite sensors for sensing-network-node applications. A front-side surface-/bulk-micromachining process on a conventional Si-substrate is developed, featuring a multifunctional SiN/poly-Si/Al layer design for diverse sensing functions. The first “pressure + acceleration + temperature + infrared” (PATIR) composite sensor with the chip size of 2.5 mm × 2.5 mm is demonstrated. Systematic theoretical design and analysis methods are developed. The diverse sensing components include a piezoresistive absolute-pressure sensor (up to 700 kPa, with a sensitivity of 49 mV/MPa under 3.3 V supplied voltage), a piezoresistive accelerometer (±10 *g*, with a sensitivity of 66 μV/*g* under 3.3 V and a −3 dB bandwidth of 780 Hz), a thermoelectric infrared detector (with a responsivity of 45 V/W and detectivity of 3.6 × 10^7^ cm·Hz^1/2^/W) and a thermistor (−25–120 °C). This design/fabrication module concept enables a low-cost monolithically-integrated “multifunctional-library” technique. It can be utilized as a customizable tool for versatile application-specific requirements, which is very useful for small-size, low-cost, large-scale sensing-network node developments.

## 1. Introduction

Over the past decade, the development of various sensing networks (e.g., wireless sensor networks, Internet-of-Things, *etc.*) has had great impact on human beings' life [1–4]. In sensing networks, sensing nodes play the key role in information gathering, and determine the performance, reliability and cost of the whole system. Along with the rapid increase of complex-environment sensing/monitoring requirements, multifunctional sensing-network nodes are in high demand [5]. Such composite sensing nodes not only monitor various objects or parameters, but also achieve better sensing characteristics by information convergence [6,7]. Although it looks straightforward to make multi-parameter sensing nodes by assembling different commercial sensors onto a single PCB circuit [8], such a PCB-based instrument unavoidably has disadvantages of big size, high power, complicated operation and high cost, limiting its application in large-scale distributed sensing networks.

Single-chip-integrated composite sensors are great candidates for the next generation of sensing nodes. By integrating various sensing components on a single chip with microelectromechanical system (MEMS) techniques, the monolithic composite sensors will feature the advantages of smaller size, lower power, and less assembly cost. By utilizing the modern IC/MEMS foundries' fabrication technologies, it will be much efficient to maximize the sensors' uniformity and minimize the product cost. Researchers have made efforts in developing sensors with combined functions [9–11]. However, the huge diversities in sensing objects (inertial, electromagnetic, thermal, *etc.*), principles (piezoresistive, capacitive, resonant, *etc.*), and applications (auto-electronics, environmental monitoring, *etc.*), have imposed great challenges in commercializing integrated composite sensor products. In addition, several developed integration techniques utilized double-sided micromachining process [12] and even silicon-on-insulator (SOI) wafers [13], which are not favorable for low-cost mass production for sensing networks. Thus, a design/fabrication platform module with low-cost integrated-process solutions is highly desirable.

In this paper, we propose a monolithic low-cost design/fabrication module with a single-sided “low-stress-silicon-nitride (LS-SiN)/polysilicon (poly-Si)/Al” process on conventional substrates. The versatile SiN/poly-Si/Al layers realize diverse sensing functions. A “pressure + acceleration + temperature + infrared” (PATIR) prototype chip for sensing node applications is demonstrated. In the platform module, diverse sensing structure designs are monolithically integrated by a compatible process in which the fabrication steps and materials are shared. More importantly, the proposed design/fabrication module concept will lead to a low-cost single-chip-integrated “function library”, as a customizable tool for the customers. Users may activate or de-activate these functions using a hybrid-packaged circuit chip. As a result, it will remarkably save the design/fabrication/packaging costs for versatile multifunctional sensing-network nodes. This paper is organized as follows: Section 2 describes the proposed design strategy for the composite sensor. Section 3 presents the structure and parameter design of the prototype PATIR sensor. Section 4 depicts the developed single-sided low-cost fabrication module. Section 5 shows the measurement results of the PATIR sensor, followed by the conclusions in Section 6.

## 2. Design Strategy for Monolithic Composite Sensor

To form a low-cost design/fabrication platform module for a multifunctional composite sensor, the device structure, material and fabrication method should be comprehensively considered and selected. The objective single-chip-integrated “function library” will contain different sensing components with various structure types and shared materials. The key is to develop a low-cost, highly compatible process module for versatile structure and material design.

### 2.1. Structure Compatibility Design

**Figure 1.**
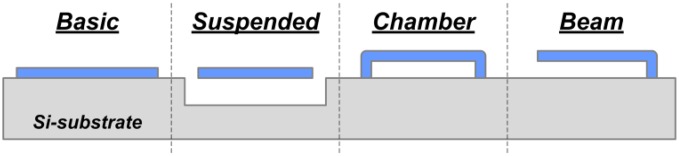
Schematic of four sensing-structure types (cross-section view).

We may divide the common sensor structures into four essential categories, including the basic type, suspended type, chamber type and beam type, as shown in Figure 1. The basic type refers to the sensing structures fabricated directly on (or in) the substrate, such as the thermistor (with insulating layer), PN junction, and so on. The suspended type depicts certain structures that need to be physically isolated from the substrate, such as thermal elements with a large isolation gap. The chamber type represents the sensor structures with a sealed cavity, like the microfluidic chamber/channels, the reference-pressure chamber, *etc.* The beam type expresses the sensor series with movable suspended beam-based structures, such as many inertial sensors (e.g., accelerometers).

To form these structure types in Figure 1, two fabrication methods can be utilized, *i.e.*, bulk micromachining and surface micromachining. For the suspended structure that requires large gap from the substrate, bulk micromachining, which can form a large cavity in the Si substrate by a dry or wet etching method is preferred. The bulk process is also used to make the sealed chamber type structure. A backside etching is first conducted to form a cavity while leaving a thin membrane at the front-side. Then a wafer bonding to the backside is implemented to finally seal the cavity [14]. To avoid non-uniform etching depth from the backside in batch wafer process, SOI wafers are utilized, in which the buried oxide layer acts as a built-in etch-stopping layer [13]. However, backside processing plus wafer bonding on SOI wafer will increase the foundry fabrication cost dramatically. In contrast, the surface micromachining is much more suitable for making the chamber and beam type structures. In surface micromachining, a sacrificial layer is utilized to form the chamber, which can deliver exact and tunable gap dimensions [15]. Material deposition (e.g., poly-Si, TEOS) can be used to plug the release holes of the sacrificial layer and form a sealed chamber [16]. All the processes in surface micromachining are conducted from the front-side, which is favorable for MEMS foundries. Thus in our developed design/fabrication platform module, we propose to fabricate the sealed chamber and beam type structures by a surface micromachining method. To obtain the suspended structure over a deep cavity, we may use front-side bulk micromachining at the last step of the fabrication flow. This design method avoids SOI wafer and double-sided process, therefore, cost-efficient composite sensors can be implemented.

### 2.2. Material Compatibility Design

For a compatible and simplified design/fabrication module, materials should be selected for a maximized versatility with proper mechanical/electrical properties. To form the critical structure layer as shown in Figure 1 (in blue), a material featuring eminent mechanical strength is needed. Among the commonly used IC/MEMS materials (e.g., SiO_2_, SiN, poly-Si), poly-Si shows good mechanical properties. However, the poly-Si structures should be protected well in any Si-etching process, which may increase the process complexity. SiO_2_ features intrinsic compressive stress, which may induce buckling effects and lead to fragile structures. In contrast, the low-stress SiN features intrinsic tensile stress and high fracture strength, which is suitable for mechanical structures. Additionally, SiN shows a low coefficient of thermal expansion, moderate thermal conductivity and high infrared absorptivity. Therefore the SiN is chosen to form the force-sensing beams/diaphragms, electrical/thermal insulating layers and infrared absorbing layers. To form the sensing components, poly-Si is selected due to its wide conductivity and piezoresistivity tunability. It can be utilized to form piezoresistors, magnetic sensing components with Hall effect, and thermistors. For metal interconnections, Al is adopted. In addition, the poly-Si/Al pair is a good thermocouple for relative temperature sensing. In summary, we propose a SiN/poly-Si/Al multilayer technique for various sensing applications.

### 2.3. Design/Fabrication Module Demonstration: PATIR Sensor

**Figure 2.**
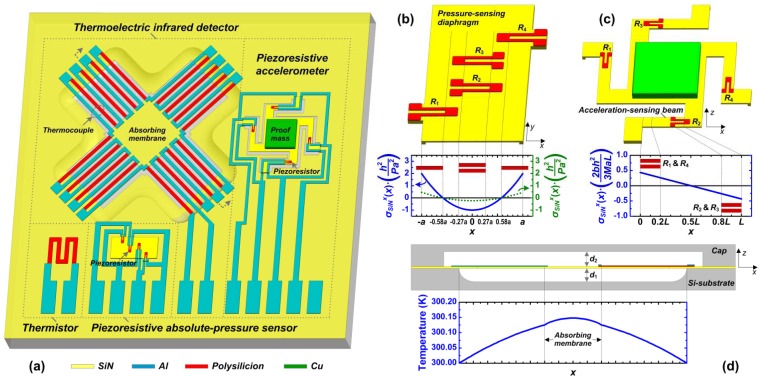
(**a**) Schematic of the prototype “pressure + acceleration + temperature + infrared” (PATIR) composite sensor design (cap not shown here). (**b**) The absolute-pressure sensor with the stress-distribution in pressure-sensing diaphragm. (**c**) The accelerometer with stress-distribution analysis along the beam. (**d**) The infrared detector (cross-section view) with simulated temperature distribution.

To demonstrate the proposed design/fabrication module with single-sided SiN/poly-Si/Al process, we developed a monolithic PATIR composite sensor, which consists of a piezoresistive absolute pressure sensor, a piezoresistive accelerometer, a thermistor and a thermoelectric infrared detector. In terms of structure features, these four sensing components correspond to the chamber type, beam type, basic type and suspended type structures, respectively. In terms of sensing functions, the pressure sensor monitors the absolute pressure (up to 700 kPa), and is able to sense multiple environment parameters, like a change of altitude. The accelerometer detects the acceleration from environmental vibration or movement (0–±10 *g*), which can also be used as a movement-activated “waking-up” component for the whole system. The infrared detector monitors the thermal radiation change from the environment, such as human body invasion or hazard gas leakage. The thermistor is to monitor the reference temperature of the environment (−25–120 °C), which is critical for calibrating the devices' temperature drifts. Such a “4-in-1” composite sensor can be employed in buildings and traffic facilities for environment monitoring, hazard warning and security management. The designed sensor structure is shown in Figure 2(a). The absolute-pressure sensor features a sealed quasi-vacuum chamber on Si-substrate with SiN structure diaphragm and poly-Si piezoresistors. The accelerometer utilizes the SiN beams, SiN support layer with Cu mass and poly-Si piezoresistors. The infrared detector adopts suspended SiN absorbing layer and poly-Si/Al thermocouples over the concave cavity. The thermistor is made of poly-Si strips on SiN insulating layer. Design details of these four sensing components are in Section 3.

## 3. Composite Sensor Design

### 3.1. Piezoresistive Absolute-Pressure Sensor

The designed absolute-pressure sensor is based on a vacuum chamber with four Wheatstone-bridge piezoresistors placed on the pressure-sensing diaphragm, as shown in Figure 2(b). When a pressure is applied on the SiN diaphragm, the diaphragm deflects and induces stress distribution. By designing a rectangular diaphragm with high length-to-width ratio, the stress distribution close to the center is quasi-one-dimensional, of which the stress along width direction (*x*-), *σ*_SiN_*^x^* (*x*), is much significant than that in the length direction (*y*-), *σ*_SiN_*^y^* (*x*), which can be expressed as [17]:

(1) $${\sigma_{\text{SiN}}}^{x}\left(x\right)=\frac{P}{h^{2}}\left(3x^{2}-a^{2}\right)$$

(2) $${\sigma_{\text{SiN}}}^{y}\left(x\right)=\nu_{\text{SiN}}\frac{P}{h^{2}}\left(3x^{2}-a^{2}\right)$$

where *P* the is applied pressure, *a* is the half width of the diaphragm, *h* is the diaphragm thickness, and *ν*_SiN_ is the Poisson's ratio of SiN. The stress distributions are shown in Figure 2(b). Four meander-shape piezoresistors are designed, with their longitudinal parts placed along the *x*-direction at the high-stress area for large sensitivity. Then the sensitivity is calculated as [15]:

(3) $$\begin{array}{ll} S=\frac{V_{\text{out}}}{P} & =\frac{1}{2}\left|\frac{\Delta R_{1}}{R_{1}}-\frac{\Delta R_{2}}{R_{2}}\right|\frac{V_{\text{in}}}{P}\\ & \approx \frac{a^{2}}{h^{2}}\frac{E_{\text{Si}}/\left(1-{\nu_{\text{Si}}}^{2}\right)}{E_{\text{SiN}}/\left(1-{\nu_{\text{SiN}}}^{2}\right)}V_{\text{in}}\cdot \left[\frac{0.83\left(\pi_{L}+\pi_{T}\nu_{\text{SiN}}\right)R_{L}-0.17\left(\pi_{L}\nu_{\text{SiN}}+\pi_{T}\right)R_{T}}{R_{L}+R_{T}}\right] \end{array}$$

where *V*_in_ and *V*_out_ are the supplied voltage and signal output of the Wheatstone bridge, *E*_SiN_ and *E*_Si_ are the Young's modulus of SiN and poly-Si, *R*_L_ and *R*_T_ represents the total resistance of the longitudital parts and the turning parts of one piezoresistor, *π*_L_ and *π*_T_ depicts the longitudinal and transversal piezoresistive coefficient of poly-Si, respectively.

The designed structure parameters and material properties for the absolute-pressure sensor are summarized in Table 1. The calculated sensitivity from Equation (3) is 112 mV/MPa (with supplied voltage *V*_in_ = 3.3 V). Due to the residual in-plane tensile stress of the deposited LS-SiN diaphragm (about 100 MPa), the stiffness of the diaphragm will be increased and therefore the real sensitivity will be reduced from Equation (3) [17]. Finite element analysis (FEA) by ANSYS shows that the sensor's sensitivity is 55 mV/MPa with considering the effect of residual stress (see Supplementary Information).

The surface micromachining techniques are utilized for the structure formation. The pressure chamber is constructed by sacrificial-layer releasing and quasi-vacuum sealing, which is described in Section 4.

### 3.2. Piezoresistive Accelerometer

The designed accelerometer is designed to measure the acceleration perpendicular to the substrate. The structure is composed of a proof-mass and four folded beams with Wheatstone-bridge piezoresistors, as shown in Figure 2(c). It is a rotating type of “double-clamped beam + central mass” design, which effectively releases the tensile stress in the deposited LS-SiN thin-film, and therefore prevents the sensitivity degradation caused by the residual stress. Comparing to the free-end cantilevers, the double-clamped beam shows smaller deflection, which may fit well with the small gap from surface micromachining. When the acceleration along z-direction is applied, the proof-mass moves and induces stress distribution on the beams. By designing a slender beam with high length-to-width ratio, the stress distribution becomes quasi-one-dimensional along the length direction. Take one beam in Figure 2(c) as an example, with considering the material's residual stress, the longitudinal (*x*-) stress *σ*_SiN_*^x^* (*x*) can be expressed as [17]:

(4) $${\sigma_{\text{SiN}}}^{x}\left(x\right)=\frac{3Ma}{2bh^{2}}\left(\frac{1}{2}L-x\right){\left(1+\gamma_{1}\frac{\sigma_{0}L^{2}}{E_{\text{SiN}}h^{2}}\right)}^{-1}$$

where *a* is the applied acceleration, *M* represents the proof-mass, *L*, *b* and *h* depict the beam length, width and thickness, respectively, *γ*_1_ (=0.295) is a coefficient expressing the additional contribution of the axial force to the beam stiffness, and *σ*_0_ is the residual tensile stress in the SiN beam. Then the sensitivity is calculated as [15]:

(5) $$\begin{array}{ll} S=\frac{V_{\text{out}}}{a} & =\frac{1}{2}\left|\frac{\Delta R_{1}}{R_{1}}-\frac{\Delta R_{2}}{R_{2}}\right|\frac{V_{\text{in}}}{a}\\ & \approx \frac{ML}{bh^{2}}\frac{E_{\text{Si}}/\left(1-{\nu_{\text{Si}}}^{2}\right)}{E_{\text{SiN}}/\left(1-{\nu_{\text{SiN}}}^{2}\right)}V_{\text{in}}\cdot \left(\frac{0.6R_{L}\pi_{T}+0.2R_{T}\pi_{T}}{R_{L}+R_{T}}\right){\left(1+\gamma_{1}\frac{\sigma_{0}L^{2}}{E_{\text{SiN}}h^{2}}\right)}^{-1} \end{array}$$

A set of designed structure parameters of the accelerometer is shown in Table 2. The material properties are the same as in Table 1. The suspended rotating structure design can release the deposition stress (100 MPa) significantly. FEA simulation shows that the residual stress *σ*_0_ at piezoresistor location is lowered to only about 5 MPa (see Supplementary Information). Then the calculated sensitivity *S* is 69 μV/*g* (*V*_in_ = 3.3 V).

To realize the accelerometer structure, a similar SiN chamber to the absolute-pressure sensor is firstly formed, and then Cu-electroplating is implemented to obtain the proof-mass, followed by SiN patterning for the beam structures, as detailed in Section 4.

### 3.3. Thermoelectric Infrared Detector

The designed thermoelectric infrared detector consists of a SiN infrared absorbing membrane and a set of poly-Si/Al thermocouples connected in series. The absorbing membrane and the main parts of the thermocouples are suspended from the Si-substrate with a deep etching cavity, to ensure good thermal insulation from the bulk-Si heat sink. The thermocouples' hot-ends contact with the absorber, and its cold-ends are anchored to the Si-substrate. By absorbing the infrared radiation from the detected object with high temperature, the absorber causes a temperature increase at the hot junctions. According to the Seebeck effect, this temperature difference (Δ*T*_HC_) between the hot-ends and cold-ends will induce a voltage output *V*_out_ from the thermopile. Then the dectector's responsivity *R*_v_, can be calculated as [20]:

(6) $$R_{v}=\frac{V_{\text{out}}}{\phi_{0}A}=\frac{N\alpha_{12}\Delta T_{\text{HC}}}{\phi_{0}A}=\frac{N\alpha_{12}\eta }{K_{\text{th}}}$$

where *Φ*_0_ is the infrared radiation flux, *A* expresses the absorber area, *N* is the number of thermocouples, *α*_12_ is the temperature-correlated Seebeck coefficient of the thermocouple, *η* is the absorptivity of the absorbing membrane, *K*_th_ is the thermocouples' thermal conductance. Another important performance parameter, the normalized detectivity D* is calculated as:

(7) $$D^{*}=\frac{\sqrt{A}}{v_{n}\;/\;R_{v}}=R_{v}\sqrt{\frac{A}{4k_{B}T_{0}R}}$$

where *v*_n_ is the detector's Johnson–Nyquist noise, *k*_B_ is the Boltzmann constant, *T*_0_ is the reference temperature, *R* is the thermocouples' electrical resistance. The thermal conductance *K*_th_ mainly contains three parts that exist between the absorber and heat sink, expressed as:

(8) $$K_{\text{th}}=K_{\text{stru}}+K_{\text{gas}}+K_{\text{rad}}$$

where *K*_stru_, *K*_gas_ and *K*_rad_ represent the thermal conductance of the thermocouple structure, thermal conductance of the atmospheric gas and radiation, respectively, as shown in Figure 2(d). *K*_stru_ consists of the thermal conductance of the poly-Si, Al and dielectric, depicted as [21]:

(9) $$K_{\text{stru}}=N\frac{k_{1}t_{1}w_{1}}{l_{1}}+N\frac{k_{2}t_{2}w_{2}}{l_{2}}+\frac{k_{3}t_{3}w_{3}}{l_{3}}$$

where *k*_n_ is the material's thermal conductivity, *t*_n_, *w*_n_ and *l*_n_ represent the effective thickness, width and length of the layer (n = 1, 2, 3 for the poly-Si, Al and dielectric, respectively. The thermal conductance of the atmospheric gas is [20]:

(10) $$K_{\text{gas}}=w_{3}l_{3}h_{\text{gas}}=w_{3}l_{3}k_{\text{gas}}\left(\frac{1}{d_{1}}+\frac{1}{d_{2}}\right)$$

where *h*_gas_ and *k*_gas_ is the convection coefficient and thermal conductivity of the atmospheric gas, respectively, *d*_1_ is the distance between the absorbing membrane and the etching cavity's bottom, *d*_2_ is the distance between the membrane and the top cap wafer. The radiation-induced thermal conductance is (assuming Δ*T*_HC_ ≪ *T*_0_):

(11) $$K_{\text{rad}}=4\xi_{\text{eff}}w_{3}l_{3}\sigma {T_{0}}^{3}$$

where *ξ*_eff_ is the effective emissivity coefficient of the thermopile structure, *σ* is the Stefan-Boltzmann constant.

Apparently, the keys to improve the infrared detector's performance are to achieve high Δ*T*_HC_ and small *K*_th_. Thus a long slender thermocouple and deep bulk-Si etching are preferred for the detector design. In addition, by reducing the resistance *R*, the resistor's thermal noise can be effectively reduced for high detectivity. Circular and square absorbing membranes are designed, respectively. The two sets of designed parameters and material properties are summarized in Table 3. In the thermocouple design of square-membrane device, the poly-Si and Al strips are laterally laid, while in the circular-membrane sensor, the poly-Si and Al are vertically overlapped with the insulating layer. FEA simulation by ANSYS shows the temperature distribution pattern along the square-membrane device structure, as shown in Figure 2(d). With a radiation-density of 6.5 mW/cm^2^ (the same as the value in experiment as described in Section 5.3), a temperature difference Δ*T*_HC_ of 0.12 °C is obtained between the two ends of thermocouples (see Supplementary Information). In the simulation, the membrane is assumed to absorb all the radiation without reflection or transmission. Then the calculated responsivity *R*_v_ is 64 V/W and the detectivity D* is 3.8 × 10^7^ cm·Hz^1/2^/W. For circular-membrane detector, the simulated *R*_v_ and D* are 69 V/W and 5.4 × 10^7^ cm·Hz^1/2^/W, respectively.

To form the suspended infrared detector structure, a front-side bulk-Si etching process is utilized after the thermocouple patterning, as described in Section 4.

### 3.4. Thermistor

A poly-Si resistor is designed as the thermistor to detect the environment temperature *T*. The sensitivity is expressed as:

(12) $$S=\frac{R-R_{0}}{T-T_{0}}=TCR\cdot R_{0}$$

where *R* is the measured resistance at ambitious temperature *T*, *R*_0_ is the resistance at the reference temperature *T*_0_, *TCR* is the temperature coefficient of resistance. A meander-shape thermistor is designed, with length of 1,500 μm, width of 25 μm and *R*_0_ of 6 kΩ.

## 4. Microfabrication Technique

**Figure 3.**
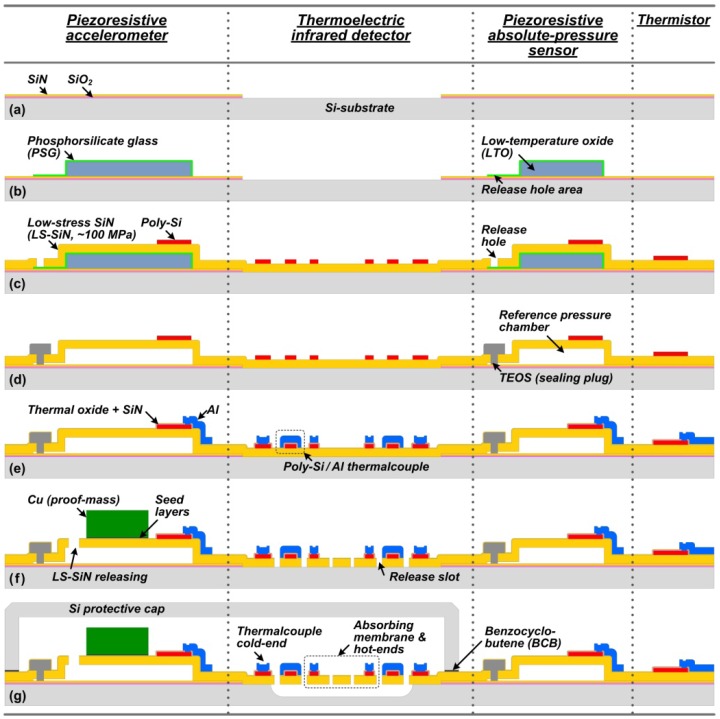
The developed single-side-integrated process flow for the prototype PATIR composite sensor.

A front-side surface-/bulk-micromachining process module on conventional Si-substrate is developed for the monolithic integrated PATIR sensor. Various sensing structures with multifunctional SiN/poly-Si/Al layers design are implemented for diverse sensing functions. The chip fabrication and wafer-level prepackaging flow is shown in Figure 3 and detailed as follows.

- The fabrication process starts from a conventional 4 inches (100) Si-wafer (0–10 Ω·cm). The substrate is firstly doped by boron iron (B^+^) implantation, with energy of 80 keV and dose of 8 × 10^15^ cm^−2^. The doping is to turn the substrate into a very conductive electrode for the accelerometer's self-test function (see Section 5.2). Then the substrate is annealed in an O_2_ atmosphere at 1,100 °C for 65 min, to activate the impurity doping and simultaneously obtain a 0.5 μm thick thermal oxide layer. Next, a 0.5 μm thick low stress SiN film is deposited by low pressure chemical vapor deposition (LPCVD). The as-fabricated SiO_2_/SiN films act as electrical insulating layer for the pressure sensor, accelerometer and thermistor. At the infrared detector part, the SiO_2_/SiN films are removed by reactive ion etching (RIE), to reduce the thickness of the ultimate absorbing membrane.
- A 2 μm thick low temperature oxide (LTO) and a 0.25 μm thick phosphor silicate glass (PSG) are deposited by LPCVD and patterned in turn. The PSG/LTO layers serve as the sacrificial structures for the pressure sensor and accelerometer. The LTO defines the gap between the function layer and the substrate. The PSG layer covering LTO is to fasten the lateral etching speed, in which the phosphorous content determines the etching rate. The lateral release holes are only composed of PSG for easy sealing in later process.
- A 1.2 μm thick LS-SiN is deposited by LPCVD at 875 °C. This multifunctional layer serves as force-sensing diaphragm/beam in the pressure sensor and accelerometer, as well as the absorbing membrane in the infrared detector. By adjusting the flow rates of gases, the silicon-rich SiN film is tuned to reach a low residual tensile-stress of about 100 MPa, measured by a laser scanning profilemeter. A too high residual stress would degrade the sensitivity of pressure sensor and accelerometer. On the other hand, the stress should not be too low for mechanical and chemical stability. Then a 0.8 μm thick fine-grained poly-Si is deposited by LPCVD at 620 °C, followed by B+ implantation (80 keV, 8 × 10^15^ cm^−2^). The poly-Si layer is then patterned to form the piezoresistors, the thermocouple part and the thermistor. Next, the 1.2 μm thick LS-SiN film is patterned by RIE for the first time to open the release holes for sacrificial-layer etching.
- The sacrificial LTO layer is removed by 40% HF for 8–10 min. The etching progress can be inspected under a microscope via the transparent SiN diaphragm. Though HF has a high etching selectivity between LTO and LS-SiN of over 100:1, the distribution of release holes is carefully designed to realize a uniform etching inside the complex structures. Then 1.4 μm thick tetraethyl orthosilicate (TEOS) is deposited by LPCVD, and patterned by buffered oxide etch (BOE) to seal the release holes. The reasons for choosing TEOS as the sealing plugs include: (1) the TEOS provides an excellent conformal coverage at the step of release holes, (2) the low vacuum during TEOS deposition (50 Pa at 720 °C) vacuums the pressure sensor's reference chamber simultaneously [23]. As the wafer is cooled down to room temperature (25 °C), the reference pressure is further lowered to about 15 Pa (100 mtorr).
- By annealing in O_2_ atmosphere at 1,100 °C for 1 h, a uniform ion distribution is obtained in poly-Si resistors with the sheet resistance of 100 Ω/sq. Simultaneously, 0.4 μm thick thermal oxide is obtained outside the poly-Si. Then a 0.2 μm thick LS-SiN is deposited by LPCVD. The “oxide + SiN” layers are utilized as dual protection on the poly-Si structures in the following bulk-Si etching. Next, the contact holes are opened by RIE. A 0.6 μm thick Al layer is sputtered and patterned. This Al layer serves as the interconnection and the thermocouple part for the infrared detector. At this step, the processes on pressure sensor and thermistor are completed.
- A seed layer of 0.05 μm thick Ti/W and 0.3 μm thick Cu is sputtered, followed by a 10 μm thick Cu electroplating for the proof-mass of the accelerometer. Then the seed layer is removed by ammonium persulfate (APS). The Cu-mass also acts as an electrode for the accelerometer's self-test function (see Section 5.2) by contacting with one Al pad. Then the LS-SiN is patterned by RIE for the second time to release the proof-mass and SiN suspending beams, and then the fabrication of the accelerometer is completed. Meanwhile, the release slots for the infrared detector are opened.
- After the above surface-micromachining steps, a bulk-micromachining is utilized to remove the Si-substrate beneath the infrared detector's absorbing membrane and thermocouples, for thermal insulation. The bulk-Si is then etched by XeF_2_ isotropic dry etching process, with XeF_2_ of 2 torr, N_2_ of 60 torr, each cycle of 20 s for 800 cycles in total. The etching depth is about 50 μm. Then the infrared detector is finished at this step. The XeF_2_ etches SiN and SiO_2_ much slower than bulk-Si, with the selectivity of over 1:200 and 1:1,000, respectively. Thus the fabricated “0.4 μm thick oxide + 0.2 μm thick SiN” layers from (e) protect the poly-Si strips very well. Finally, a cap-wafer with RIE-processed cavities are fabricated, coated by 2 μm thick benzocyclobutene (BCB) and bonded to the sensor wafer. This wafer-level prepackaging secures the accelerometer and infrared detectors during the following saw-dicing process. The pressure sensor and the thermistor are laid outside of the cap wafer.

**Figure 4.**
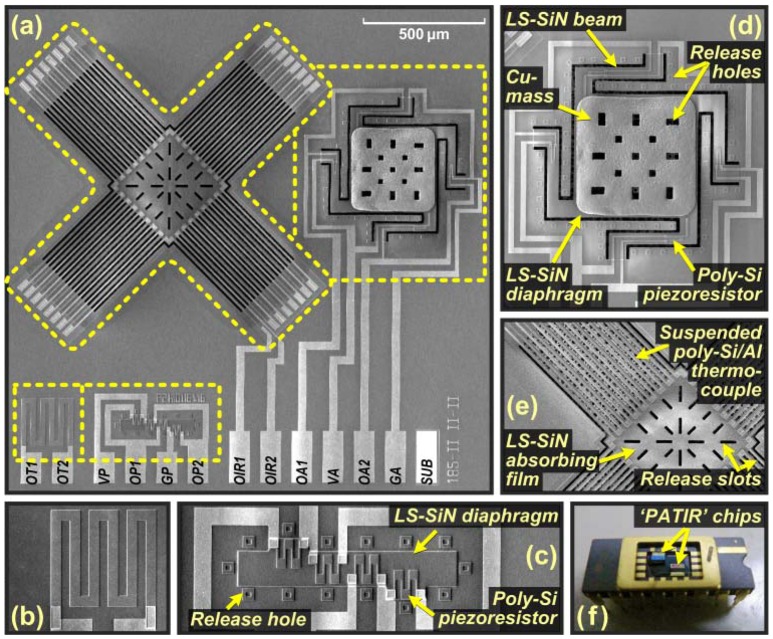
Photos showing the prototype PATIR composite sensor: (**a**) and (**f**) full views, (**b**) thermistor, (**c**) piezoresistive absolute-pressure sensor, (**d**) piezoresistive accelerometer, (**e**) thermoelectric infrared detector.

After the saw-dicing, the sensor chips are wire-bonded for the second-stage packaging and testing. Figure 4 shows the photos of the fabricated PATIR sensor, with a chip size of 2.5 mm × 2.5 mm. The developed process module enables large-scale fabrication for the composite sensor. For the sensing node applications, the signal processing circuit chip could be fabricated separately to avoid involving deep etching process into IC fabrication and ensure a high yield. The sensor chip and IC chip would be packaged together by a hybrid method such as system-in-package (SIP), which is our ongoing work.

## 5. Results

**Figure 5.**
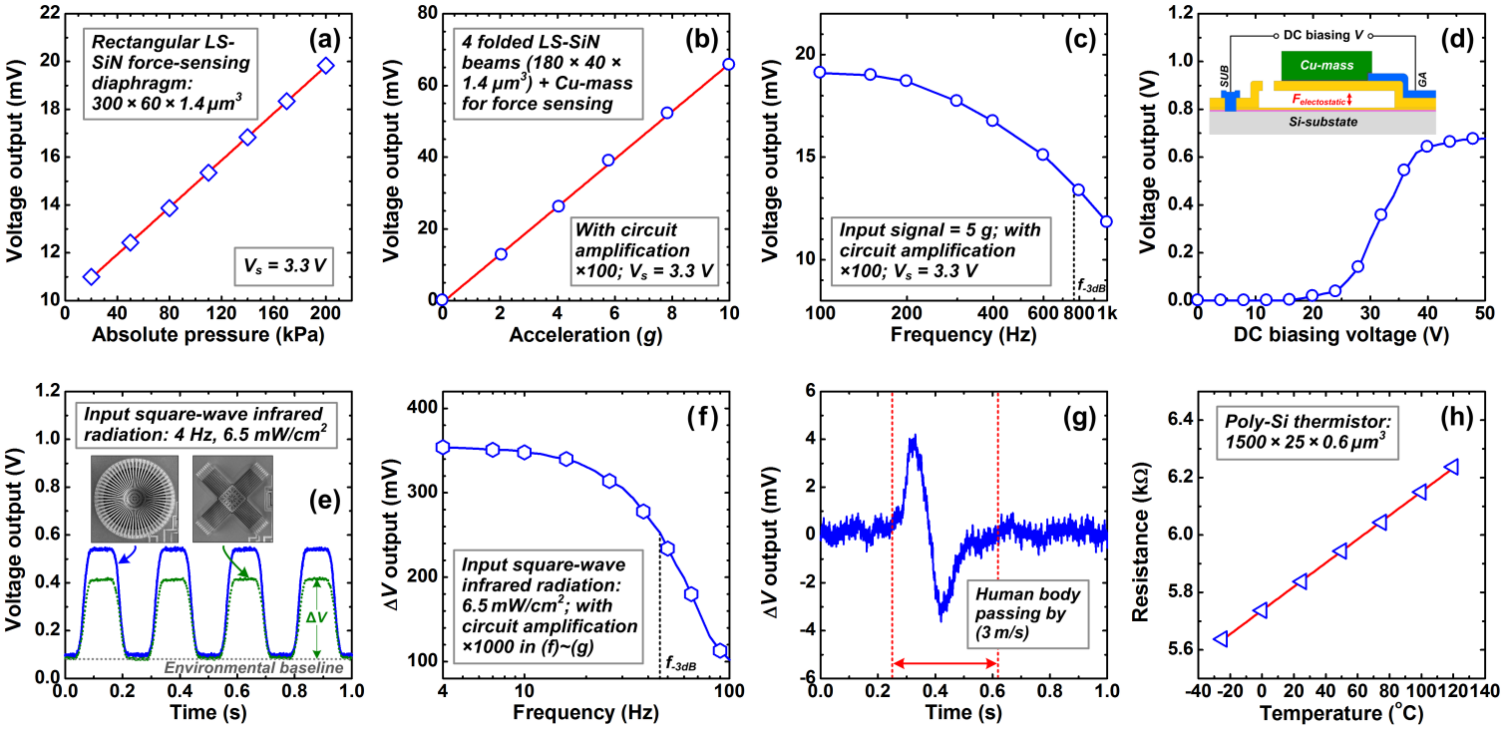
Measurement results of the PATIR composite sensor. (**a**) Output response of the absolute-pressure sensor under pressure inputs. (**b**) Output response of the accelerometer under acceleration loads. (**c**) Frequency response of the accelerometer. (**d**) Self-test response of the accelerometer. The inset illustrates the principle of the developed self-test technique. (**e**) Real-time responses of the circular-membrane and square-membrane infrared detectors under the blackbody radiation signal. (**f**) Frequency response of the circular-membrane infrared detector. (**g**) Real-time response of the circular-membrane infrared detector when a human body passing by with 2 m distance. (**h**) Output response of the thermistor under temperature inputs.

The various sensing components of the PATIR sensor are characterized. The device responses are measured separately by using different test apparatus, without involving the cross interference among the sensing components. The measured results are shown in Figure 5.

### 5.1. Pressure sensor

For pressure sensing characterization, the sensor is sealed in a metal-can package with gas path connected to a hand-pump (GE Druck PV411). By tuning the hand-pump with a standard pressure gauge (GE Druck DPI 104, with accuracy of ±0.05%), the pressure varies and the pressure sensor's Wheatstone-bridge-output is monitored.

The measured output response to the applied pressure at room temperature is shown in Figure 5(a). The pressure sensor shows a linear sensitivity of 49 mV/MPa (with supplied voltage *V*_in_ = 3.3V), which is very close to the simulated result. In the ranges of 20–200 kPa and 20–700 kPa, the measured non-linearity are ±0.50% FSO and ±1.2% FSO, respectively. A six-point three-cycle reciprocating test in 20–200 kPa shows that the sensor's repeatability error, hysteresis error and accuracy are 0.21% FSO, 0.52% FSO and ±0.75% FSO, respectively.

### 5.2. Accelerometer

For acceleration sensing measurement, the sensor is mounted on a vibration shaker (JZK-5, with ±10 *g* range from 20 Hz to 5 kHz). The sensor's Wheatstone-bridge-output during the vibration is amplified by a voltage amplifier (Endevco DC Amplifier 136) and then read by a digital oscilloscope (Tektronix TDS3014B, Beaverton, USA, with 9 bits). A standard vibration transducer (Brüel & Kjær 8305-001, Nærum, Denmark, with accuracy of ±0.5%) is mounted on the vibration shaker for the measurement of applied acceleration waves.

The measured output response to the applied acceleration at 100 Hz is shown in Figure 5(b). In the range of 0–10 *g*, the accelerometer sensor shows a sensitivity of 66 μV/*g* (*V*_in_ = 3.3V) and a non-linearity of ±0.41% FSO. The results match very well with the theoretical analysis. Figure 5(c) shows the accelerometer's frequency response curve, measured by keeping the applied acceleration at 5 *g*. It shows that the −3 dB frequency of the accelerometer is 780 Hz. As mentioned in Section 4, the substrate and the proof-mass form a parallel capacitor from the developed process module. This design enables a wafer-level on-line self-test technique for the low-cost quality-inspection in the volume fabrication. By supplying a DC sweeping voltage on this parallel capacitor (*i.e.*, via the pads “GA” and “SUB” in Figure 4), the electrostatic force induces deflection of the SiN beams and therefore the piezoresistive outputs, as shown in Figure 5(d). The sample shows a quasi-step-shape response, with linear output under small voltage while a significant increase at large voltage due to pull-in effect.

### 5.3. Infrared Detector

For infrared detecting characterization, the sensor is exposed to the infrared radiation from a 733 K blackbody source (IRCON BCL-08C-1, Santa Cruz, USA) with a mechanical chopper. The electrical output of the detector is amplified and read by the digital oscilloscope. The infrared radiation source generates a power density of 6.5 mW/cm^2^ over the detector, with a distance of 15 cm.

Figure 5(e) shows the measured real-time output waveforms of the detectors with circular and square membranes under the 4-Hz-chopped blackbody's radiation. It shows a responsivity of 45 V/W and a detectivity of 3.6 × 10^7^ cm·Hz^1/2^/W for the circular-membrane device, and a responsivity of 41 V/W as well as a detectivity of 2.4 × 10^7^ cm·Hz^1/2^/W for the square-membrane detector. Comparing to the simulated results, a 35% reduction of *R*_v_ or D* is observed, indicating the energy dissipation due to the membrane's reflection/transmission as well as the absorption of air. Figure 5(f) shows the circular-membrane detector's frequency response curve. A −3 dB frequency of 46 Hz is obtained. As a demonstration of the sensing application, a real-time test on the passing human body with 2 m distance is carried out, as shown in Figure 5(g).

### 5.4. Thermistor

For temperature sensing measurement, the thermistor is placed in a thermostat-controlled oven (WCT-1P-C). The sensor's resistance is monitored while changing the temperature from −25 °C to 120 °C. The measured curve of resistance *versus* temperature is shown in Figure 5(h). The measured *TCR* and sensitivity are 7.1 × 10^−4^/°C and 4.1 Ω/°C, respectively, with a non-linearity of ±0.71% FSO.

### 5.5. Comparison with Reported Composite Sensors

Table 4 compares the PATIR sensor with several published composite sensors. Our process module features four sensing functions, conventional (100) Si-substrate, single-sided process with small chip size. The measured performance of PATIR sensor compares favorably to reported devices.

## 6. Conclusions

A new design/fabrication platform module with low-cost process solutions for a monolithic-integrated composite sensor is reported. A single-sided surface-/bulk-micromachining process on conventional Si-substrate is developed, featuring versatile “SiN/poly-Si/Al” layers. The first “pressure + acceleration + temperature + infrared” (PATIR) composite sensor with a chip size 2.5 mm × 2.5 mm is demonstrated. Detailed design and analysis the sensors are conducted. The measured performances cover multiple requirements in the Internet-of-Things applications. The developed design/fabrication module is very promising for small-size, low-cost easy-to-customize sensing networks.

## Supplementary Material



## Figures and Tables

**Table 1.** Structure parameters and material properties of the designed piezoresistive absolute-pressure sensor.

**Parameter**	**Value**
Diaphragm thickness *h* (μm)	1.4
Diaphragm width 2*a* (μm)	60
Diaphragm length 2*b* (μm)	360
Piezoresistor thickness (μm)	0.6
Number of piezoresistor's longitudinal parts	4
Longitudinal part length (μm)	16
Longitudinal part width (μm)	4
Turning part size (μm)	16
Resistance (kΩ)	2
Young's modulus of SiN *E*_SiN_ (GPa)	224 [18]
Young's modulus of poly-Si *E*_Si_ (GPa)	160 [18]
Poisson's ratio of SiN *ν*_SiN_	0.23 [18]
Poisson's ratio of poly-Si *ν*_Si_	0.23 [18]
Longitudinal piezoresistive coefficient of poly-Si *π*_L_ (10^−10^ Pa^−1^)	1.56 [19]
Transverse piezoresistive coefficient of poly-Si *π*_T_ (10^−10^ Pa^−1^)	−0.44 [19]

**Table 2.** Structure parameters of the designed piezoresistive accelerometer.

**Parameter**	**Value**
Beam thickness *h* (μm)	1.4
Beam width *b* (μm)	40
Beam length *L* (μm)	180
Piezoresistor thickness (μm)	0.6
Number of piezoresistor's longitudinal parts	2
Longitudinal part length (μm)	36
Longitudinal part width (μm)	4
Turning part size (μm)	14
Resistance (kΩ)	2
Proof-mass *M* (ng)	12

**Table 3.** Structure parameters and material properties of the designed thermoelectric infrared detector.

**Parameter**	**Value**
Absorbing membrane thickness (μm)	1.4
Absorbing membrane area *A* (mm^2^)	0.16 (square), 0.13 (circular)
Poly-Si thickness *t*_1_ (μm)	0.6
Al thickness *t*_2_ (μm)	0.8
Dielectric thickness *t*_3_ (μm)	1.2
Air gap *d*_1,2_ (μm)	50
Number of thermocouples	32 (square), 60 (circular)
Thermocouple length *l*_1,2_ (μm)	600 (square), 520 (circular)
Thermocouple width *w*_1,2_ (μm)	8 (square), 22 (circular, averaged)
Resistance (kΩ)	240 (square), 140 (circular)
Thermal conductivity of SiN (W·m^−1^·K^−1^)	15 [21]
Thermal conductivity of poly-Si (W·m^−1^·K^−1^)	29 [21]
Thermal conductivity of Al (W·m^−1^·K^−1^)	238 [21]
Thermal conductivity of Air (W·m^−1^·K^−1^)	0.083 [22]

**Table 4.** Comparison of PATIR sensor with reported single-chip integrated composite sensors.

**Sensor**	**Size (mm × mm)**	**Number of function**	**Process**	**Sensitivity of pressure sensor (mV/MPa)** *	**Sensitivity of accelerometer (μV/*g*)** *	**Detectivity of infrared detector (cm·Hz^1/2^/W)**	**Sensitivity of thermistor (Ω/°C)**
This work	2.5 × 2.5	4	(100) wafer, single-sided	49	66	3.6 × 10^7^	4.1
[24]	3.0 × 3.0	1 †	(100) wafer, single-sided	—	—	4.5 × 10^7^	—
[25]	1.6 × 1.6	2	(100) wafer, single-sided	80	30	—	—
[26]	2.5 × 2.5	2	(111) wafer, single-sided	108	100	—	—
[27]	4.0 × 6.0	3	SOI wafer, double-sided	66	46	—	5.6

* Source voltage *V*_in_ = 3.3 V;

† Integrated with signal processing circuits.
