# Supplementary material for: Monolithic Composite “Pressure + Acceleration + Temperature + Infrared” Sensor Using a Versatile Single-Sided “SiN/Poly-Si/Al” Process-Module
Source: Sensors (Basel). 2013 Jan 16;13(1):1085–101. doi: 10.3390/s130101085 (PMC3574723; doi:10.3390/s130101085)
Supplement: Supplementary file 1 [file sensors-13-01085-s001.pdf]

OPEN ACCESS
sensors
ISSN 1424-8220
www.mdpi.com/journal/sensors

Supplementary Information

Monolithic Composite "Pressure + Acceleration + Temperature + Infrared" Sensor Using a Versatile Single-Sided "SiN/Poly-Si/Al" Process-Module. *Sensors* 2013, *13*, 1085-1101

Zao Ni, Chen Yang *, Dehui Xu, Hong Zhou, Wei Zhou, Tie Li, Bin Xiong and Xinxin Li *

State Key Laboratory of Transducer Technology, Shanghai Institute of Microsystem and Information Technology, Chinese Academy of Sciences, 865 Changning Road, Shanghai 200050, China; E-Mails: nizao@mail.sim.ac.cn (Z.N.); dehuixu@mail.sim.ac.cn (D.X.); zhouhong@mail.sim.ac.cn (H.Z.); zhouwei@mail.sim.ac.cn (W.Z.); tli@mail.sim.ac.cn (T.L.); bxiong@mail.sim.ac.cn (B.X.)

* Authors to whom correspondence should be addressed; E-Mails: cyang@mail.sim.ac.cn (C.Y.); xxli@mail.sim.ac.cn (X.L.); Tel.: +86-21-6251-1070; Fax: +86-21-6213-1744.

1. Theoretical Design and Finite Element Analysis on Absolute-Pressure Sensor

1.1. Structure Design

Figure S1(a) illustrates the pressure-sensing SiN diaphragm in the absolute-pressure sensor. The diaphragm length, width and thickness are symbolized as 2b, 2a and h, respectively. Assuming $2b \gg 2a \gg h$, the deflection and stress along the x-direction are [1]:

$$w_{\mathrm{SiN}}(x) = \frac{P}{2E_{\mathrm{eff}}h^3}x^2(a-x)^2 = \frac{P(1-\nu_{\mathrm{SiN}}{}^2)}{2E_{\mathrm{SiN}}h^3}x^2(a-x)^2 \quad \text{S(1.1)}$$

$$\sigma_{\mathrm{SiN}}{}^x(x) = \frac{P}{h^2}(3x^2-a^2) \quad \text{S(1.2)}$$

$$\sigma_{\mathrm{SiN}}{}^y(x) = \nu_{\mathrm{SiN}}\frac{P}{h^2}(3x^2-a^2) \quad \text{S(1.3)}$$

where x varies in ($-a$, a), P is the applied pressure, ν_{SiN} is the Poission's ratio, E_{eff} is the effective Young's module:

$$E_{\mathrm{eff}} = \frac{E_{\mathrm{SiN}}}{1-\nu_{\mathrm{SiN}}{}^2} \quad \text{S(1.4)}$$

The stress distribution is shown in Figure S1(b).

Figure S1. (**a**) Schematic of the rectangular pressure-sensing diaphragm and piezoresistors of the absolute-pressure sensor. (**b**) Stress distribution in the diaphragm and the piezoresistors' longitudinal-parts placement design.

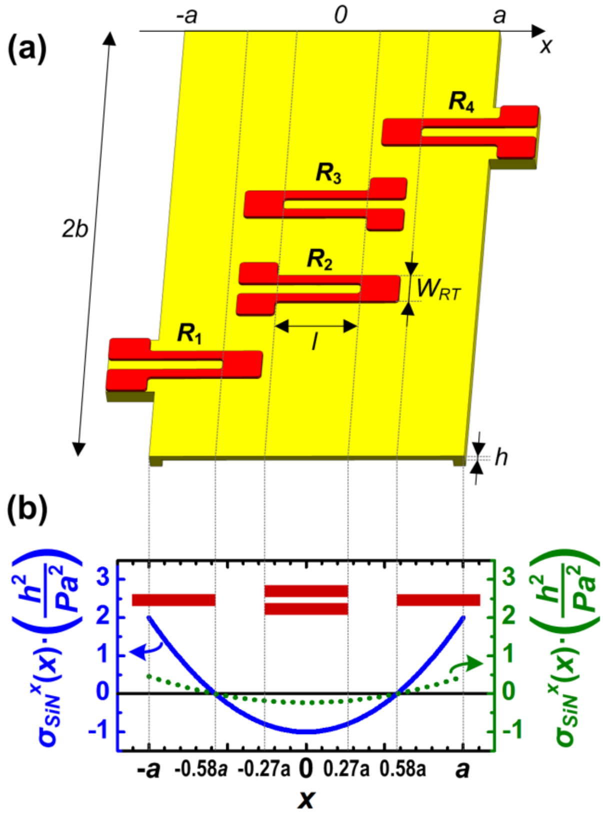

To realize a Wheatstone-bridge piezoresistive sensing, four meander-shape poly-Si piezoresistors $R_1 \sim R_4$ are designed, with their longitudinal parts placed along the x-direction at the high-stress area for large sensitivity, as shown Figure S1(b). In detail, the length of tensile-stress area is $0.42a$. Thus the longitudinal parts of R_1 and R_4 are laid at the tensile-stress areas of $(-a, -0.58a)$ and $(0.58a, a)$. Note that the length of longitudinal part l is bigger than $0.42a$, therefore a minor part is laid outside the $(-a, a)$ region. In contrast, both longitudinal parts of R_2 and R_3 are placed at the compressive-stress area of $(-l/2, l/2)$. Each piezoresistor has n longitudinal segments, and $(1-n)$ turning parts in which the current flows transversally. The total resistance, longitudinal resistance and effective transverse resistance of one piezoresistor are symbolized as R, R_L and R_T, respectively. Thus, $R = R_L + R_T$.

Since the strain of poly-Si strips are nearly the same as the strain of SiN, *i.e.*,

$$\varepsilon_{\mathrm{Si}} = \frac{\sigma_{\mathrm{Si}}(x)}{E_{\mathrm{Si}} / \left(1-\nu_{\mathrm{Si}}^{2}\right)} \approx \varepsilon_{\mathrm{SiN}} = \frac{\sigma_{\mathrm{SiN}}(x)}{E_{\mathrm{SiN}} / \left(1-\nu_{\mathrm{SiN}}^{2}\right)} \qquad \text{S(1.5)}$$

We obtain

$$\sigma_{\mathrm{Si}}(x) \approx \sigma_{\mathrm{SiN}}(x) \frac{E_{\mathrm{Si}} / \left(1-\nu_{\mathrm{Si}}^{2}\right)}{E_{\mathrm{SiN}} / \left(1-\nu_{\mathrm{SiN}}^{2}\right)} \qquad \text{S(1.6)}$$

Thus the stress in the poly-Si piezoresistor will be

$$\sigma_{\mathrm{Si}}^{\ x}(x)=\frac{P}{h^2}\left(3x^2-a^2\right)\frac{E_{\mathrm{Si}}\big/\left(1-\nu_{\mathrm{Si}}^{\ 2}\right)}{E_{\mathrm{SiN}}\big/\left(1-\nu_{\mathrm{SiN}}^{\ 2}\right)} \tag{S(1.7)}$$

$$\sigma_{\mathrm{Si}}^{\ y}(x)=\nu_{\mathrm{SiN}}\frac{P}{h^2}\left(3x^2-a^2\right)\frac{E_{\mathrm{Si}}\big/\left(1-\nu_{\mathrm{Si}}^{\ 2}\right)}{E_{\mathrm{SiN}}\big/\left(1-\nu_{\mathrm{SiN}}^{\ 2}\right)} \tag{S(1.8)}$$

1.2. Performance Analysis

From the above discussion, we can calculate the pressure-induced resistance change of the piezoresistors. For R_1 and R_4,

$$\begin{aligned}
&\Delta R_1=\Delta R_4\\
&=\left(\frac{0.42a}{l}\right)R_{\mathrm{L}}\left(\pi_{\mathrm{L}}\frac{\int_{-a}^{-0.58a}\sigma_{\mathrm{Si}}^{\ x}(x)\,dx}{0.42a}+\pi_{\mathrm{T}}\frac{\int_{-a}^{-0.58a}\sigma_{\mathrm{Si}}^{\ y}(x)\,dx}{0.42a}\right)\\
&+\left(\frac{n/2}{1-n}\right)R_{\mathrm{T}}\left(\pi_{\mathrm{L}}\frac{\int_{-0.58a}^{-0.58a+W_{\mathrm{RT}}}\sigma_{\mathrm{Si}}^{\ y}(x)\,dx}{W_{\mathrm{RT}}}+\pi_{\mathrm{T}}\frac{\int_{-0.58a}^{-0.58a+W_{\mathrm{RT}}}\sigma_{\mathrm{Si}}^{\ x}(x)\,dx}{W_{\mathrm{RT}}}\right)\\
&=\left[\frac{0.39a}{l}R_{\mathrm{L}}\left(\pi_{\mathrm{L}}+\nu_{\mathrm{SiN}}\pi_{\mathrm{T}}\right)-\left(\frac{n/2}{1-n}\right)\left(\frac{W_{\mathrm{RT}}^{\ 2}}{a^2}-\frac{1.74W_{\mathrm{RT}}}{a}+0.0092\right)R_{\mathrm{T}}\left(\nu_{\mathrm{SiN}}\pi_{\mathrm{L}}+\pi_{\mathrm{T}}\right)\right]\\
&\cdot\frac{Pa^2}{h^2}\frac{E_{\mathrm{Si}}\big/\left(1-\nu_{\mathrm{Si}}^{\ 2}\right)}{E_{\mathrm{SiN}}\big/\left(1-\nu_{\mathrm{SiN}}^{\ 2}\right)}
\end{aligned} \tag{S(1.9)}$$

For R_2 and R_3,

$$\begin{aligned}
&\Delta R_2=\Delta R_3\\
&=R_{\mathrm{L}}\left(\pi_{\mathrm{L}}\frac{\int_{-0.5l}^{0.5l}T_{\mathrm{Si}}^{\ x}(x)\,dx}{l}+\pi_{\mathrm{T}}\frac{\int_{-0.5l}^{0.5l}T_{\mathrm{Si}}^{\ y}(x)\,dx}{l}\right)\\
&+R_{\mathrm{T}}\left(\pi_{\mathrm{L}}\frac{\int_{-0.5l-W_{\mathrm{RT}}}^{-0.5l}T_{\mathrm{Si}}^{\ y}(x)\,dx}{W_{\mathrm{RT}}}+\pi_{\mathrm{T}}\frac{\int_{-0.5l-W_{\mathrm{RT}}}^{-0.5l}T_{\mathrm{Si}}^{\ x}(x)\,dx}{W_{\mathrm{RT}}}\right)\\
&=-\left[\left(1-\frac{l^2}{4a^2}\right)R_{\mathrm{L}}\left(\pi_{\mathrm{L}}+\nu_{\mathrm{SiN}}\pi_{\mathrm{T}}\right)-\left(1-\frac{3lW_{\mathrm{RT}}}{2a^2}-\frac{3l^2}{4a^2}-\frac{W_{\mathrm{RT}}^{\ 2}}{a^2}\right)R_{\mathrm{T}}\left(\nu_{\mathrm{SiN}}\pi_{\mathrm{L}}+\pi_{\mathrm{T}}\right)\right]\\
&\cdot\frac{Pa^2}{h^2}\cdot\frac{E_{\mathrm{Si}}\big/\left(1-\nu_{\mathrm{Si}}^{\ 2}\right)}{E_{\mathrm{SiN}}\big/\left(1-\nu_{\mathrm{SiN}}^{\ 2}\right)}
\end{aligned} \tag{S(1.10)}$$

Thus the Wheatstone-bridge output voltage turns to be

$$\begin{aligned}
V_{\mathrm{out}}&=V_{\mathrm{in}}\left|\frac{R_4+\Delta R_4}{R_3+\Delta R_3+R_4+\Delta R_4}-\frac{R_2+\Delta R_2}{R_1+\Delta R_1+R_2+\Delta R_2}\right|\approx V_{\mathrm{in}}\frac{\left|\Delta R_1-\Delta R_2\right|}{2R}\\
&=\frac{V_{\mathrm{in}}}{2\left(R_{\mathrm{L}}+R_{\mathrm{T}}\right)}\cdot\left[\alpha R_{\mathrm{L}}\left(\pi_{\mathrm{L}}+\nu_{\mathrm{SiN}}\pi_{\mathrm{T}}\right)-\beta R_{\mathrm{T}}\left(\nu_{\mathrm{SiN}}\pi_{\mathrm{L}}+\pi_{\mathrm{T}}\right)\right]\cdot\frac{Pa^2}{h^2}\frac{E_{\mathrm{Si}}\big/\left(1-\nu_{\mathrm{Si}}^{\ 2}\right)}{E_{\mathrm{SiN}}\big/\left(1-\nu_{\mathrm{SiN}}^{\ 2}\right)}
\end{aligned} \tag{S(1.11)}$$

where

$$\alpha = 1 + \frac{0.39a}{l} - \frac{l^2}{4a^2},$$
$$\beta = \left(\frac{n/2}{1-n}\right)\left(\frac{W_{\mathrm{RT}}{}^2}{a^2} - \frac{1.74W_{\mathrm{RT}}}{a} + 0.0092\right) - \left(1 - \frac{3lW_{\mathrm{RT}}}{2a^2} - \frac{3l^2}{4a^2} - \frac{W_{\mathrm{RT}}{}^2}{a^2}\right) \qquad \text{S(1.12)}$$

Then the sensitivity is calculated as

$$S = \frac{V_{\mathrm{out}}}{P} = \frac{1}{2}\frac{a^2}{h^2}\frac{E_{\mathrm{Si}}/\left(1-\nu_{\mathrm{Si}}{}^2\right)}{E_{\mathrm{SiN}}/\left(1-\nu_{\mathrm{SiN}}{}^2\right)}V_{\mathrm{in}} \cdot \left[\frac{\alpha R_{\mathrm{L}}\left(\pi_{\mathrm{L}} + \nu_{\mathrm{SiN}}\pi_{\mathrm{T}}\right) - \beta R_{\mathrm{T}}\left(\nu_{\mathrm{SiN}}\pi_{\mathrm{L}} + \pi_{\mathrm{T}}\right)}{R_{\mathrm{L}} + R_{\mathrm{T}}}\right] \qquad \text{S(1.13)}$$

Based on the design parameters in this paper, we obtain

$$S = \frac{a^2}{h^2}\frac{E_{\mathrm{Si}}/\left(1-\nu_{\mathrm{Si}}{}^2\right)}{E_{\mathrm{SiN}}/\left(1-\nu_{\mathrm{SiN}}{}^2\right)}V_{\mathrm{in}}\left[\frac{0.83R_{\mathrm{L}}\left(\pi_{\mathrm{L}} + \nu_{\mathrm{SiN}}\pi_{\mathrm{T}}\right) - 0.17R_{\mathrm{T}}\left(\nu_{\mathrm{SiN}}\pi_{\mathrm{L}} + \pi_{\mathrm{T}}\right)}{R_{\mathrm{L}} + R_{\mathrm{T}}}\right] \qquad \text{S(1.14)}$$

The calculated sensitivity is 112 mV/MPa (V_{in} = 3.3 V). Equation (1.14) gives a design guideline on the rectangular-diaphragm based pressure sensor, in which the effects of various structure and material parameters on the sensor performance are revealed.

Due to the residual in-plane tensile stress of the deposited LS-SiN diaphragm (about 100 MPa), the stiffness of the diaphragm will be increased and therefore the real sensitivity will be reduced. Then the ANSYS numerical analysis is implemented with considering the effect of residual tensile stress.

Figure S2 illustrates the simulated stress distribution of $\sigma_{\mathrm{SiN}}{}^x(x)$ (with deflected shape), with 1 MPa pressure perpendicular to the diaphragm and an in-plane 100 MPa tensile stress. The $\sigma_{\mathrm{SiN}}{}^x(x)$ curve along x-direction is shown in Figure S3(a). In contrast, the simulated $\sigma_{\mathrm{SiN}}{}^x(x)$ distribution without considering the residual tensile stress is also shown in Figure S3(b).

Figure S2. Simulated stress distribution of pressure-sensing diaphragm under 1 MPa applied pressure.

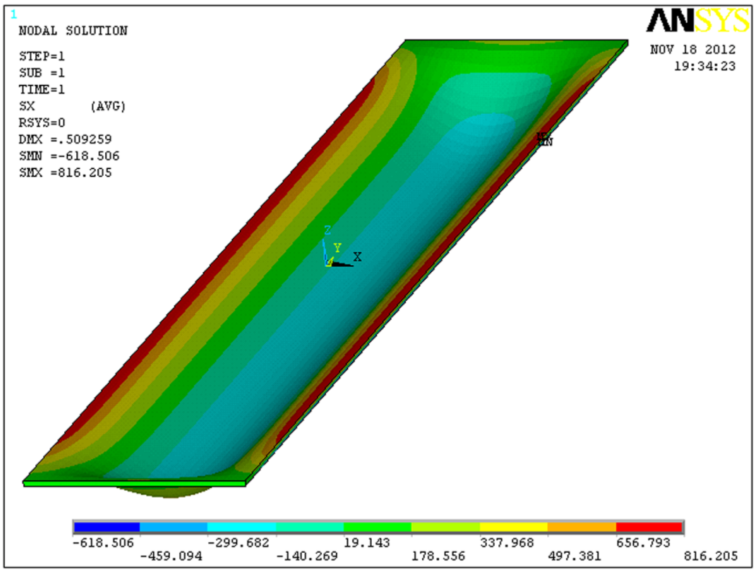

Figure S3. Simulated stress distributions at the pressure-sensing diaphragm surface. (**a**) Residual tensile stress considered. (**b**) Residual tensile stress neglected.

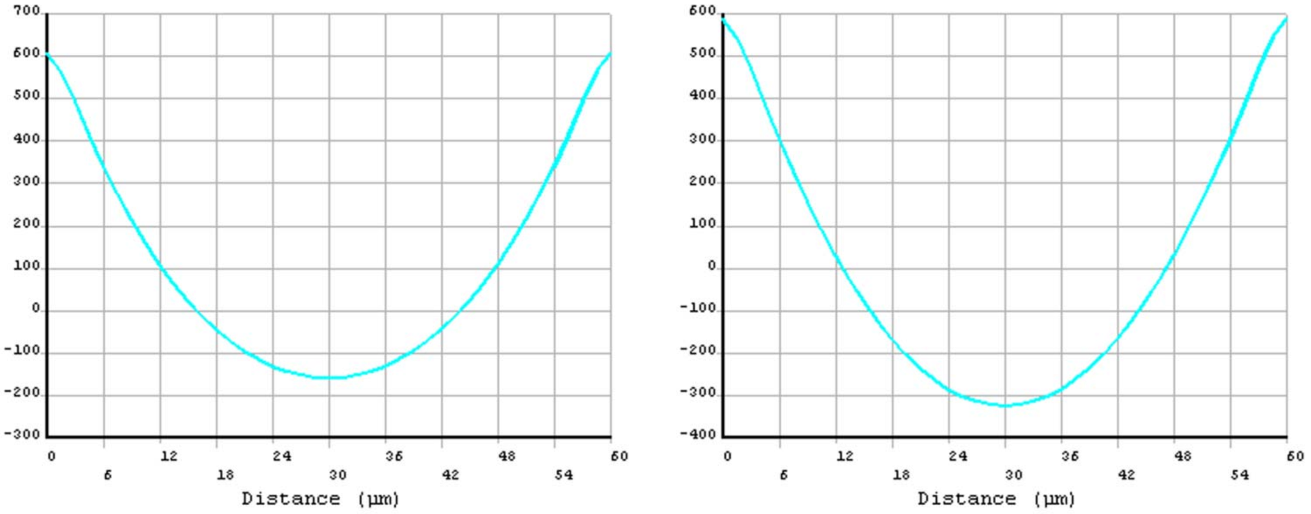

(**a**) (**b**)

From the simulated stress data in Figure S3, the resistance changes (ΔR_1 and ΔR_2) are calculated according to (1.9) and (1.10). Note that the stress data in Figure S3(a) is firstly subtracted by 100 MPa before the calculation. Then we obtain the simulated sensitivity of the pressure sensor. With the supplied voltage (V_{in}) of 3.3 V, the sensitivity of the device without residual tensile stress is 71 mV/MPa. By considering the residual stress, the simulated sensitivity decreases to 55 mV/MPa.

2. Theoretical Analysis and Finite Element Analysis on Accelerometer

2.1. Structure Design

Figure S4(a) illustrates the structure design of the accelerometer. The length, width and thickness of the force-sensing beam are symbolized as L, b and h, respectively. With considering the residual stress, the deflection and stress in the beam along the x-direction are [1]:

$$w_{\mathrm{SiN}}(x) = \frac{Ma}{2E_{\mathrm{SiN}}bh^3}x^2\left(\frac{3}{2}L - x\right)\left(1+\gamma_1\frac{\sigma_0 L^2}{E_{\mathrm{SiN}}h^2}\right)^{-1} \quad \text{S(2.1)}$$

$$\sigma_{\mathrm{SiN}}{}^{x}(x) = \frac{3Ma}{2bh^2}\left(\frac{1}{2}L - x\right)\left(1+\gamma_1\frac{\sigma_0 L^2}{E_{\mathrm{SiN}}h^2}\right)^{-1} \quad \text{S(2.2)}$$

where x varies in (0, L), a is the applied acceleration, v_{SiN} is the Poission's ratio, E_{sin} is the Young's module of SiN, γ_1 (=0.295) is a coefficient expressing the additional contribution of the axial force to the beam stiffness, σ_0 is the residual tensile stress in the LS-SiN beam. As the beam is free along the y-direction, $\sigma_{\mathrm{SiN}}{}^{y}(x)$ is ignored. The stress distribution is shown in Figure S4(b).

Figure S4. (**a**) Schematic of structure design of the accelerometer. (**b**) Stress distribution in the beam and the piezoresistors' longitudinal-parts placement design.

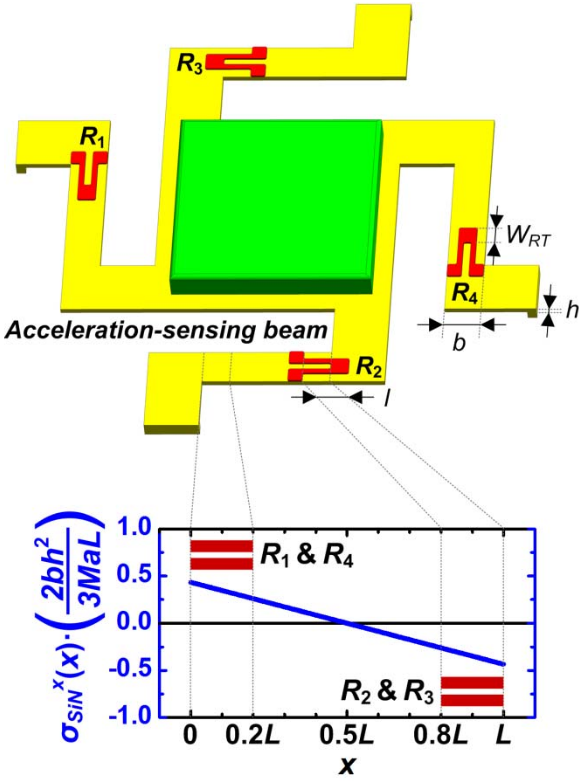

To realize a Wheatstone-bridge piezoresistive sensing, four poly-Si piezoresistors R_1 ~ R_4 are designed, with their longitudinal parts placed along the x-direction at the high-stress area for large sensitivity, as shown Figure S4(b). In detail, the length of tensile-stress area is 0.5L. Thus the longitudinal parts of R_1 and R_4 are laid at the tensile-stress areas of (0, l). In contrast, both longitudinal parts of R_2 and R_3 are placed at the compressive-stress area of ($L-l$, L). Each piezoresistor has two longitudinal segments, as well as one square turning part (size = W_{RT}) in which the current flows transversally. The total resistance, longitudinal resistance and transverse resistance of one piezoresistor are symbolized as R, R_L and R_T, respectively. Thus, $R = R_L + R_T$.

With the similar analysis in Part 1, we obtain the strain in poly-Si strips as

$$\sigma_{Si}^{\ x}(x) = \frac{3Ma}{2bh^2}\left(\frac{1}{2}L - x\right)\frac{E_{Si}/\left(1-\nu_{Si}^{\ 2}\right)}{E_{SiN}/\left(1-\nu_{SiN}^{\ 2}\right)}\left(1+\gamma_1\frac{\sigma_0 L^2}{E_{SiN}h^2}\right)^{-1} \qquad \text{S(2.3)}$$

2.2. Performance Analysis

From the above discussion, we can calculate the pressure-induced resistance change of the piezoresistors. For R_1 and R_4,

$$\Delta R_1=\Delta R_4=R_{\mathrm{L}}\pi_{\mathrm{L}}\frac{\int_0^l \sigma_{\mathrm{Si}}{}^x(x)dx}{l}+R_{\mathrm{T}}\pi_{\mathrm{T}}\frac{\int_l^{l+W_{\mathrm{RT}}} \sigma_{\mathrm{Si}}{}^x(x)dx}{W_{\mathrm{RT}}}$$
$$=\left[\left(\frac{L-l}{2}\right)R_{\mathrm{L}}\pi_{\mathrm{L}}+\left(\frac{L-2l-W_{\mathrm{RT}}}{2}\right)R_{\mathrm{T}}\pi_{\mathrm{T}}\right]\cdot\frac{3Ma}{2bh^2}\frac{E_{\mathrm{Si}}/(1-\nu_{\mathrm{Si}}{}^2)}{E_{\mathrm{SiN}}/(1-\nu_{\mathrm{SiN}}{}^2)}\left(1+\gamma_1\frac{\sigma_0L^2}{E_{\mathrm{SiN}}h^2}\right)^{-1} \quad \text{S(2.4)}$$

For R_2 and R_3,

$$\Delta R_2=\Delta R_3=R_{\mathrm{L}}\pi_{\mathrm{L}}\frac{\int_{L-l}^{L} \sigma_{\mathrm{Si}}{}^x(x)dx}{l}$$
$$=-\left(\frac{L-l}{2}\right)R_{\mathrm{L}}\pi_{\mathrm{L}}\cdot\frac{3Ma}{2bh^2}\frac{E_{\mathrm{Si}}/(1-\nu_{\mathrm{Si}}{}^2)}{E_{\mathrm{SiN}}/(1-\nu_{\mathrm{SiN}}{}^2)}\left(1+\gamma_1\frac{\sigma_0L^2}{E_{\mathrm{SiN}}h^2}\right)^{-1} \quad \text{S(2.5)}$$

Thus the sensitivity is calculated as

$$S=\frac{V_{\mathrm{out}}}{a}\approx\frac{V_{\mathrm{in}}}{a}\frac{|\Delta R_1-\Delta R_2|}{2R}=V_{\mathrm{in}}\cdot\left[\frac{(L-l)R_{\mathrm{L}}\pi_{\mathrm{L}}+(0.5L-l-0.5W_{\mathrm{RT}})R_{\mathrm{T}}\pi_{\mathrm{T}}}{R_{\mathrm{L}}+R_{\mathrm{T}}}\right]\cdot\frac{3M}{4bh^2}\frac{E_{\mathrm{Si}}/(1-\nu_{\mathrm{Si}}{}^2)}{E_{\mathrm{SiN}}/(1-\nu_{\mathrm{SiN}}{}^2)}\left(1+\gamma_1\frac{\sigma_0L^2}{E_{\mathrm{SiN}}h^2}\right)^{-1} \quad \text{S(2.6)}$$

Based on the design parameters in this paper, we obtain

$$S=\frac{V_{\mathrm{out}}}{a}=\frac{ML}{bh^2}\frac{E_{\mathrm{Si}}/(1-\nu_{\mathrm{Si}}{}^2)}{E_{\mathrm{SiN}}/(1-\nu_{\mathrm{SiN}}{}^2)}V_{\mathrm{in}}\left(\frac{0.6R_{\mathrm{L}}\pi_{\mathrm{L}}+0.2R_{\mathrm{T}}\pi_{\mathrm{T}}}{R_{\mathrm{L}}+R_T}\right)\left(1+\gamma_1\frac{\sigma_0L^2}{E_{\mathrm{SiN}}h^2}\right)^{-1} \quad \text{S(2.7)}$$

For the residual stress, ANSYS numerical analysis is conducted, as show in Figure S5. It shows that the rotating structure design releases the deposition stress significantly, and the residual stress σ_0 at piezoresistor location is lowered to only about 5 MPa. Then the calculated sensitivity of accelerometer is 69 μV/*g*.

Figure S5. Simulated residual stress distribution in the beam of accelerometer.

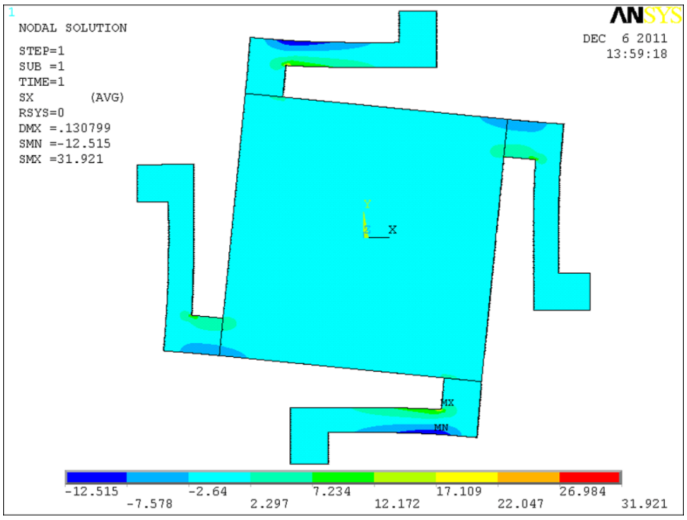

3. Finite Element Analysis on Infrared Detector

Numerical simulation by using ANSYS is conducted to simulate the temperature distribution over the structure of infrared detector under radiation. With the applied radiation-density of 6.5 mW/cm^2, the temperature distribution at one-quarter of the structure are shown in Figures S6 and S7. Then we obtain that the temperature difference ΔT_{HC} between the hot and cold ends is about 0.12 °C.

Figure S6. Simulated temperature distribution over one-quarter of the infrared detector.

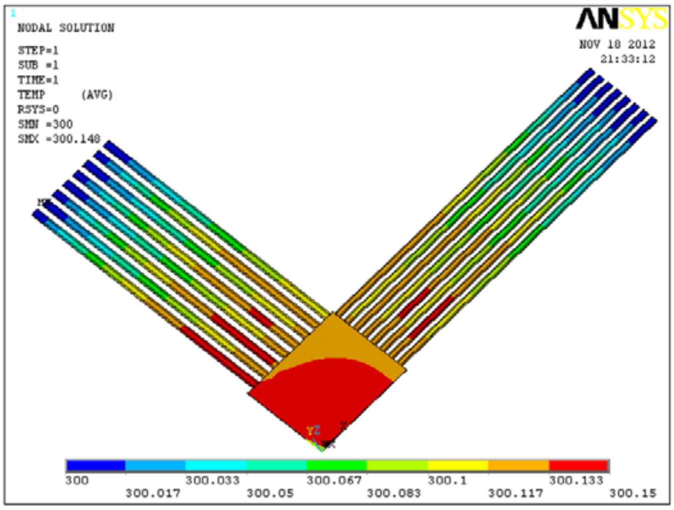

Figure S7. Simulated temperature distribution from the absorbing membrane center to the cold-end.

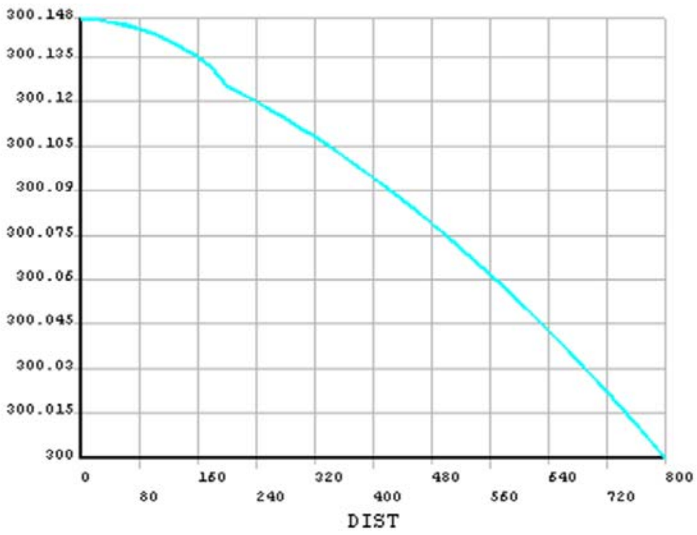

Reference

1. Bao, M. *Analysis and Design Principles of MEMS Devices*; Elsevier: Amsterdam, The Neitherlands, 2005.

© 2013 by the authors; licensee MDPI, Basel, Switzerland. This article is an open access article distributed under the terms and conditions of the Creative Commons Attribution license (http://creativecommons.org/licenses/by/3.0/).
